# Trauma exposure, PTSD and psychotic-like symptoms in post-conflict Timor Leste: an epidemiological survey

**DOI:** 10.1186/1471-244X-12-229

**Published:** 2012-12-18

**Authors:** Ian Soosay, Derrick Silove, Catherine Bateman-Steel, Zachary Steel, Paul Bebbington, Peter B Jones, Tien Chey, Lorraine Ivancic, Claire Marnane

**Affiliations:** 1 Department of Psychological Medicine, University of Auckland, Auckland, New Zealand; 2 Psychiatry Research & Teaching Unit, University of New South Wales, Liverpool Hospital, Cnr Forbes & Campbell Streets, Liverpool, Australia; 3 UCL, Mental Health Sciences Unit, Riding House Street, London, UK; 4 Department of Psychiatry, University of Cambridge, Hills Road, Cambridge, UK

## Abstract

**Background:**

Studies in developed countries indicate that psychotic-like symptoms are prevalent in the community and are related to trauma exposure and PTSD. No comparable studies have been undertaken in low-income, post-conflict countries. This study aimed to assess the prevalence of psychotic-like symptoms in conflict-affected Timor Leste and to examine whether symptoms were associated with trauma and PTSD.

**Methods:**

The Psychosis Screening Questionnaire and the Harvard Trauma Questionnaire (assessing trauma exposure and PTSD) were administered in an epidemiological survey of 1245 adults (response rate 80.6%) in a rural and an urban setting in Timor Leste. We defined PSQ screen-positive cases as those people reporting at least one psychotic-like symptom (paranoia, hallucinations, strange experiences, thought interference, hypomania).

**Results:**

The prevalence of PSQ screen-positive cases was 12 percent and these persons were more disabled. PSQ cases were more likely to reside in the urban area, experienced higher levels of trauma exposure and a greater prevalence of PTSD. PTSD only partially mediated the relationship between trauma exposure and psychotic-like symptoms.

**Conclusions:**

Psychotic-like symptoms may be prevalent in countries exposed to mass conflict. The cultural and contextual meaning of psychotic-like symptoms requires further inquiry in low-income, post-conflict settings such as Timor Leste.

## Background

Knowledge has accrued about the prevalence of psychotic-like symptoms such as hallucinations, paranoia and strange experiences in the general community [1-3]. The relatively high rates of psychotic-like experiences at a community-wide level suggests a distinction between these phenomena and clinical psychotic disorders, which have much lower prevalence rates. A clinical diagnosis of a psychotic disorder requires the simultaneous occurrence and persistence of symptoms, whereas persons experiencing isolated psychotic-like symptoms appear to be more common and are less likely to attract clinical attention [1,4].

Past studies suggest that psychotic-like symptoms are associated with being female, low levels of social support, alcohol [5] and cannabis use [6], and a family history of mental illness [5,6]. Psychotic-like symptoms appear to be more prevalent in some ethnic groups [7], but available data are restricted to minorities living in developed countries.

Trauma exposure appears to be associated with psychotic-like symptoms [8-14], with some studies indicating a dose-effect relationship [2,15]. In addition, posttraumatic stress disorder (PTSD) may mediate the relationship between trauma and psychotic-like symptoms [13].

The relationship between human rights trauma and psychotic symptoms has attracted attention for several decades. Observations following WWII suggested that psychotic-like symptoms and frank forms of psychosis were common amongst survivors of the Holocaust, although selection biases could have influenced these findings [16]. Since then, clinicians in the field of traumatic stress have argued that gross forms of human rights violations can lead to a fundamental loss of trust amongst survivors, resulting in a tendency towards suspicion and even paranoia, phenomena that are not encompassed by contemporary criteria for PTSD [17].

Little is known about the epidemiology of psychotic-like symptoms in low-income countries that are culturally distinct from nations of the west. In particular, there is a dearth of evidence about the prevalence and correlates of psychotic-like symptoms amongst populations exposed to oppression and prolonged conflict. Cultural factors need to be considered in interpreting the reporting of psychotic-like symptoms in settings such as Timor Leste, where belief in supernatural phenomena such as spirit possession is normative. The Psychosis Screening Questionnaire, the measure used in the present study, explicitly inquires whether the experience of psychotic-like symptoms reported are regarded as abnormal by other members of the society. Nevertheless, it remains possible that traditional beliefs, for example concerning curses and communication with spirits, may confound the reporting of psychotic-like symptoms. Even so, the presence of symptoms may still be clinically relevant, especially if they are associated with disability.

The present study is the first to establish the epidemiology of psychotic-like symptoms in a low-income, post-conflict society, Timor Leste. We hypothesized that psychotic-like symptoms would be common, and associated with exposure to the cumulative traumas of conflict. We also postulated that PTSD would mediate the relationship between trauma and psychotic-like symptoms.

## Methods

### Participants

The study comprised a whole population survey of persons aged 18-years or older living in two villages (sucos) in Timor Leste [18]. The Timor Leste government had undertaken a pilot census in these two areas three months prior to the present study, selecting the sites as being broadly representative of urban and rural populations in the country. The urban area, Becora, is a densely populated suburb of Dili, the capital. The rural area, Hera, situated approximately 30 minutes by road from the city, is reliant on subsistence agriculture. Data from the pilot census were used to select population tracts for inclusion in the study. Since the rate of psychotic-like symptoms was unknown, we aimed to achieve a large sample size (>1,000) by contemporary standards in the field of refugee and post-conflict mental health. We used aerial photographs and global positioning system maps produced for the government census to ensure that we identified all households in the dispersed rural area. Field workers visited houses on weekends and early in the morning on weekdays to allow employed persons ample opportunity to participate in the study. Dwellings were visited up to five times to facilitate involvement.

### Historical background

The population of Timor Leste is of mixed descent, with Malayo-Polynesian, Papuan-Melanesian, Chinese, Arabic and European contributions. The territory was severely affected by ongoing conflict during the Indonesian occupation (1975-1999) and the subsequent humanitarian crisis of 1999-2000. Extensive human rights violations were recorded during the occupation, including arbitrary detention, torture, disappearances and a number of massacres [19]. It is estimated that up to 200,000 people, about a quarter of the population at the time, died as a consequence of war and related deprivations, including famine and disease [20]. Following the fall of the Suharto regime, Indonesia agreed to a UN-sponsored referendum on independence in Timor. After a vote in favour of independence in 1999, pro-Indonesia militias initiated a campaign of violence, murder and extensive destruction of the built infrastructure of the country. Eighty percent of the population was displaced from their homes in the immediate aftermath of the vote [21]. Order was restored by the UN in 2000 and Timor Leste achieved full independence in 2002.

The areas selected for the present study were severely affected by conflict during the Indonesian occupation. The urban area was a concentration point for militants involved in the independence war, and the rural area was affected by waves of displaced people fleeing from the capital.

### Measures

#### Defining cases of psychotic-like symptoms

Psychotic-like symptoms were assessed by the Psychosis Screening Questionnaire (PSQ), an instrument used in a number of previous community studies [22]. The measure assesses the presences of five symptom domains: hypomania, thought insertion, paranoia, strange experiences and hallucinations. For each domain, a screening question establishes the presence of the symptom with additional questions then confirming that the experience is incongruent with norms in the society.

PSQ screen-positive cases were defined according to the criteria specified in the description of the measure [22], that is, endorsement of at least one of the five symptom domains and confirmation of its abnormal status following completion of the associated probes (see Table 1 for full listing of PSQ items).

**Table 1:** Frequency of psychotic-like symptoms

**PSQ domain**	**Weighted positive endorsement**	**Number of respondents (unweighted)**
**Hypomania**		
Over the past year, have there been times when you felt very happy without a break for days on end?	10.7%	128
Was there an obvious reason for this? (No)	7.5%	89
Did your relatives or friends think it was strange or complain about it?	0.2%	2
**Thought insertion**		
Over the past year, have you ever felt that your thoughts were directly interfered with or controlled by some outside force or person?	6.5%	78
Did this come about in a way that many people would find hard to believe, for instance through telepathy?	3.4%	41
**Paranoia**		
Over the past year, have there been times when you felt people were against you?	18.4%	223
Have there been times when you felt that people were deliberately acting to harm you or your interests?	9.3%	112
Have there been times when you felt that a group of people were plotting to cause you serious harm or injury?	5.4%	65
**Strange experiences**		
Over the past year, have there been times when you felt that something strange was going on?	8.1%	97
Did you feel it was so strange that other people would find it very hard to believe?	5.4%	65
**Hallucinations**		
Over the past year, have there been times when you heard or saw things that other people couldn’t?	4.3%	52
Did you at any time hear voices saying quite a few words or sentences when there was no-one around that might account for it?	2.5%	30
**Any psychotic symptom** (yes to one or more probe questions)	**29.7%**	**363**
**Met criteria based on secondary question(s)**	**12.3%**	**150**

#### PTSD

PTSD symptoms based on DSM-IV criteria were measured by the Harvard Trauma Questionnaire (HTQ) [23], the most widely applied instrument in the post-conflict and refugee field [24]. The measure has been applied in two previous studies amongst Timorese populations [25,26]. Sixteen symptom items are rated on a four point frequency scale generating a summary score (1.0 - 4.0) representing the average of the 16 items. Past psychometric studies have yielded two cut-off scores, a clinical threshold (2.5) [23] and a community threshold (2.0) [27]. In the present study we have applied the 2.0 cut-off, which in a previous analysis was identified as being associated with a high level of convergence between the HTQ PTSD scale (2.0 cut-off) and diagnoses made by medical practitioners applying the Structured Clinical Interview for DSM-IV [27], although, consistent with the past literature in the field, the questionnaire detected a wider range of persons, including some with sub-threshold symptoms [18].

#### Potentially traumatic experiences (PTEs)

The HTQ includes a potentially traumatic event (PTE) inventory of 16 common experiences of conflict-affected and refugee populations, a list that we adapted to the context of Timor-Leste. We omitted the item concerning rape following advice by community leaders that inquiry into that experience would be unacceptable in Timor. We generated an index of exposure to PTEs based on the number of categories of PTE exposure endorsed (1-2; 3-4; 5-6; 7+), demarcated to ensure sufficient representation in each grouping and excluding those with no lifetime exposure, which pertained to only a small number or participants. We selected the categorical method of analysis because the association of PTEs with mental disorder may not be linear. In the logistic regression analysis, we followed the convention of assigning the lowest trauma category (1-2) as the reference group, consistent with the literature showing an incremental increase in association between number of traumas and mental disorder [24].

We assessed the number of disability days using a measure that was applied in the Australian Bureau of Statistics national mental health survey. The measure consists of two items inquiring into the number of days in the past month that the person was (a) unable to work or perform other duties or (b) partially unable to participate in these activities, due to ill-health [28].

Socio-demographic items derived from the East Timor national census survey included age, sex, location of residence (urban/rural), education level, and employment status. Standard methods were employed to translate and back translate measures into Tetum, the local language [29].

### Training and procedure

Interviews were conducted by 20 Timorese community workers recruited via a network of non-government organizations. Four expatriate medical practitioners with experience in psychiatry and a working knowledge of Indonesian (widely spoken in Timor Leste) or Tetum (the lingua franca of the country) provided training and supervision. They were supported by bilingual interpreters. Field workers received classroom training followed by supervision in a pilot study undertaken in a village adjacent to the urban site of the main survey.

### Funding and ethics

The study was funded by the National Health and Medical Research Council of Australia, and conducted by the University of New South Wales team with the support of the Ministry of Health, Timor Leste. The research protocol conformed to the Helsinki Declaration guiding ethical research involving humans, and was approved by the Human Research Ethics Committee of the University of New South Wales, and the Timor-Leste Ministry of Health. Local leaders (chefes) also endorsed the study. Informed consent was given verbally due to low levels of literacy.

### Statistical analyses

Persons with one or more psychotic-like symptoms (designated as PSQ screen-positive cases) were compared with the remainder of the sample in univariate and multivariate logistic regression analyses, testing for associations with socio-demographic indices, PTEs (categorized as 1-2, 3-4, 5-6 and 7 or greater) and PTSD (present-absent). A further multivariate logistic regression analysis assessed disability amongst PTSD and screen-positive PSQ cases, controlling for age, location (urban/rural), education and employment. We applied simple coding for categorical and ordinal data. For dichotomous and multi-group categorical variables we identified the category with the lowest prevalence as the reference group to ensure that the odds ratios would fall in the conventional 1+ range; for ordinal variables we selected the first group as the reference category. We applied mediation analysis to test for associations involving PTE exposure, PTSD and PSQ screen-positive cases.

No adjustments were necessary for household clustering since all key indices (trauma count, PTSD, psychotic-like symptoms) yielded within-household intra-class correlations of <0.1. Regression analyses were undertaken using STATA (version 9) [30] and SAS (version 9.2) [31]. Mplus (version 6) [32] was used for the mediation analysis, applying a bootstrapping procedure involving 5000 re-samples to generate model estimates and confidence intervals. Estimated model coefficients are interpreted as Probit model coefficients [33].

## Results

The survey area included 548 dwellings housing 1544 adults. One thousand two hundred and forty-five persons participated, a response rate of 80.6%. Almost all non-responders were repeatedly absent from the house and could not be contacted. Of the whole sample, 59.0% lived in the rural area, and 51.5% were women (Table 2). Approximately two thirds (63.3%) were aged 35 years or younger. Levels of education were low, with over half (56.1%) having no or limited primary education. Subsistence agriculture was the most common occupation, with 11.2% being employed in the formal economy. Alcohol consumption was reported by 21.0%, but the amount consumed could not be accurately recorded because the most common beverage was home-made palm wine. At the time, alcohol abuse was not regarded as a problem in these communities, and illicit drug use was virtually absent.

**Table 2:** Frequencies for socio-demographic characteristics, exposure to potentially traumatic events and prevalence of PTSD

**Variable**	**Categories**	**Frequency**	**Percentage**
Age (years)			
	18-25	371	30.0
	26-35	406	33.3
	36-55	363	29.8
	56+	78	6.4
Sex			
	Female	628	51.5
	Male	592	48.5
Area of residence			
	Rural	721	59.0
	Urban	500	41.0
Education			
	None/some primary	678	56.1
	Completed primary	220	18.2
	Completed secondary	263	21.7
	Completed tertiary	50	4.1
Employment			
	Subsistence	494	40.5
	Home duties	305	25.0
	Employed	137	11.2
	Unemployed	254	21.0
Alcohol consumption			
	No	963	79.0
	Yes	256	21.0
PTE categories			
	1-2	311	25.5
	3-4	406	33.3
	5-6	285	23.4
	7+	218	17.9
PTSD positive			
	No	1158	95.0
	Yes	62	5.0

Five percent of participants exceeded the 2.0 threshold for PTSD (Table 2), and 12.3% were PSQ screen-positive cases (Table 1). Table 1 shows that paranoia (5.4%) and strange experiences (5.4%) were the most prevalent psychotic-like symptoms. Only two respondents (0.2%) reported hypomania.

In relation to PTEs, exposure to floods and earthquakes was common (76.3%), consistent with the known frequency of these events in Timor (Table 3). As expected, the majority of PTEs associated with violence and conflict occurred during the Indonesian occupation and humanitarian emergency [18]. Lack of food and water (63.0%) and burning of houses (61.5%) were common experiences. Approximately a third of participants (34.3%) had direct experiences of combat; 18.6% had been attacked or assaulted; 18.3% had witnessed a family member being murdered; and 11.2% had been tortured.

**Table 3:** Potentially traumatic events endorsed

**Lifetime trauma experience**	**Frequency**	**Percentage**
experienced flood/earthquake	931	76.3
been without food and water	769	63.0
had house burnt down	750	61.5
family member disappeared	457	37.5
experienced combat	418	34.3
been without shelter	311	25.5
been sick and unable to access medical care	272	22.3
been attacked or assaulted	227	18.6
seen family member murdered	223	18.3
family member been sick and unable to access medical care	207	17.0
had serious injury	166	13.6
seen stranger murdered	156	12.8
experienced torture	137	11.2
been forcibly separated from family	116	9.5
seen death from flood/earthquake	109	8.9
been imprisoned	84	6.9

The univariate analyses (Table 4) show that PSQ screen-positive cases were younger, tended to live in the urban area, had higher levels of education, and were either employed in the formal sector or unemployed (compared to being engaged in subsistence agriculture). Those endorsing high levels of PTEs and persons with PTSD were more likely to be PSQ screen-positive cases. In the multivariate model, PSQ screen-positive cases were more likely to be urban residents, to experience incrementally higher levels of PTE categories, and to have PTSD.

**Table 4:** Univariate and multivariate associations of socio-demographic and PTE categories for PSQ positive cases

**Variable**			**Unadjusted OR**						**Adjusted OR**				
			**OR value**		**95% CI**		**P**		**OR value**		**95% CI**		**P**
Age (years)													
	18-25		1						1				
	26-35		1.2		0.8-1.8		0.349		1.2		0.7-2.0		0.455
	36-55		0.5		0.3-0.9		0.011*		0.7		0.4-1.2		0.194
	56+		0.3		0.1-0.9		0.033*		0.4		0.1-1.4		0.142
Sex													
	Female												
	Male		1.2		0.8-1.7		0.371						
Area of residence													
	Rural												
	Urban		2.8		2.0-4.0		0.000*		2.1		1.3-3.2		0.002*
Education													
	None/some primary												
	Completed primary		1.8		1.1-2.9		0.025*		1.2		0.6-2.2		0.589
	Completed secondary		2.8		1.9-4.3		0.000*		1.7		0.9-2.9		0.088
	Completed tertiary		3.7		1.8-7.6		0.001*		1.6		0.6-4.2		0.356
Employment													
	Subsistence												
	Home duties		1.5		0.9-2.5		0.099		1.8		1.0-3.2		0.036*
	Employed		2.9		1.7-5.0		0.000*		1.1		0.6-2.2		0.771
	Unemployed		2.2		1.4-2.6		0.001*		1		0.5-1.9		0.895
Alcohol consumption													
	No												
	Yes		1.6		1.1-2.4		0.016*		1.5		1.0-2.4		0.072
PTE categories													
	1-2												
	3-4		1.6		0.8-3.2		0.167		1.7		0.9-3.4		0.135
	5-6		3.9		2.1-7.5		0.000*		3.9		2.0-7.6		0.000*
	7+		8.4		4.4-15.7		0.000*		6.9		3.4-13.7		0.000*
PTSD													
	No												
	Yes		6.1		3.5-10.5		0.000*		3.1		1.7-5.8		0.000*

Table 4 indicates that both PTSD (OR: 2.1, CI: 1.2-3.7, p=0.0105) and PSQ screen-positive cases (OR: 1.6, 95% CI: 1.1-2.5, p=0.019) were disabled with no interaction effect.

**Figure 1:**
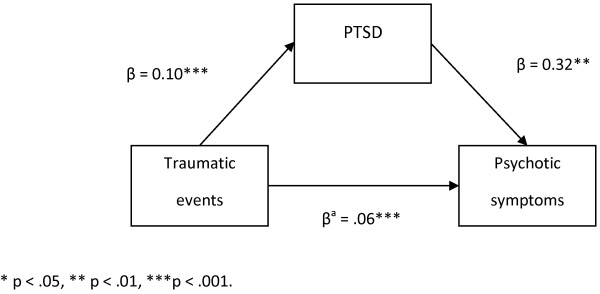
**The mediating effect of PTSD on the relationship between traumatic events and psychotic symptoms.** * p < .05, ** p < .01, ***p < .001.

Figure 1 shows the results of the mediation model. PTSD partially mediated the relationship between PTEs and PSQ screen-positive cases (effect = 0.033, 95% CI: 0.014–0.060). However, the direct effect of PTEs was comparatively greater (0.063, 95% CI: 0.034 - 0.093), indicating that the partial mediating effect of PTSD accounted for approximately 34% of the overall relationship of PTEs with PSQ screen-positive caseness.

## Discussion

To our knowledge, this is the first study documenting the population prevalence of PSQ screen-positive cases in a low-income, post-conflict society. There was a substantial prevalence of such cases, more than twice the rate for PTSD. Comparisons with previous studies need to be made with caution because of differences in sampling and indices of psychotic-like symptoms measured. Nevertheless, it is noteworthy that the rate of PSQ screen-positive cases in the British National Morbidity Survey was 4% [2]. Other studies using the same instrument in western countries have found prevalence rates ranging between 4 and 12% [34], although higher rates have been reported in studies using other measures [3]. High rates of PSQ screen-positive cases have been documented amongst ethnic minorities living in western settings. For example, amongst Romanian immigrants living in adverse social circumstances in Italy, 19% were PSQ screen-positive [34]. Similarly, a study of five ethnic groups in the UK found a higher rate of PSQ screen-positive cases in Afro-Caribbeans (12%) than in the majority Caucasian population (6%) [7]. The common characteristic of these groups is their exposure to socio-economic disadvantage, suggesting that adverse environmental conditions may be relevant in the genesis of psychotic-like symptoms.

Our findings suggested a relationship between increasing levels of PTE exposure and PSQ screen-positive cases, even after adjusting for socio-demographic variables. Persons with high levels of trauma exposure had invariably experienced extensive human rights violations such as torture, incarceration and other forms of politically motivated abuses. In that respect, our study echoes the findings of the post-WWII literature, in which associations were noted between gross forms of human rights violation and psychotic-like symptoms, for example, amongst survivors of concentration camps [16].

Recent clinical observations have supported an association between severe PTSD symptoms and psychotic-like experiences including paranoia and hallucinations [35,36]. Our analysis provided some support for the role of PTSD in mediating the link between PTEs and PSQ screen-positive cases. Nevertheless, the direct path was stronger, suggesting that, in the Timor Leste setting, PTSD played a subordinate role in mediating the effect.

The presence of psychotic-like symptoms in survivors of human rights trauma adds to the evidence supporting the recognition of a more complex form of traumatic stress [37]. Past formulations have included Disorders of Extreme Stress Not Otherwise Specified (DESNOS), considered but not included in the Diagnostic and Statistical Manual of Mental Disorders version 4 (DSM-IV) [37], and the ICD-10 category of Enduring Personality Change After Catastrophic Experiences (EPCACE) [38]. Both constellations include symptoms of mistrust, hostility and alienation, characteristics akin to paranoia and other psychotic-like symptoms.

It seems likely that exposure to prolonged persecution and conflict can attenuate the individual’s sense of trust and security, in a manner that may nevertheless be adaptive. In a minority, however, these responses may create or magnify the susceptibility to paranoia and other psychotic-like symptoms, particularly when ongoing communal divisions continue to foster fear and suspicion. The challenge is to define more clearly the boundaries between normative responses of mistrust and pathological reactions wherein suspicion and fear are transformed into frank and potentially disabling psychotic-like symptoms. In the first paper in this series, we reported estimates of clinical psychotic disorders of 1.35% for the sample as a whole, based on the Structured Clinical Interview for the DSM-IV (SCID) conducted by medical personnel amongst a subset of respondents [18]. This rate clearly is much lower than the prevalence (12.3%) of PSQ screen-positive cases reported here. Hence, as already recognised [2,18], there is a large discrepancy between PSQ screen-positive cases and the rates of clinical psychotic disorders in the population. Clinical diagnoses require the persistence of a combination of symptoms over a specified time period. In addition, the threshold for endorsing symptoms when applying screening instruments invariably is lower than that imposed by clinicians employing comprehensive clinical assessments to make formal diagnoses. In the present study, only a small number of respondents completed both the PSQ and the SCID, precluding a systematic comparison of the two forms of assessment [18]. Hence, uncertainty remains about the clinical significance of reported psychotic-like symptoms based on instruments such as the PSQ.

The PSQ explicitly inquires whether the psychotic-like experiences endorsed are regarded as alien by others living in the same setting. Nevertheless, cultural factors could still be influential in the expression of symptoms. The traditional animistic faith remains influential in Timor Leste, a system that includes beliefs about curses, being bewitched and communicating directly with ancestors. It is possible that the boundaries between these normative, cultural experiences and psychotic-like symptoms become blurred in states of distress. However, in the univariate analyses, the young, the educated and those living in the city were more likely to be PSQ screen-positive, and yet those sectors of the population are more likely to have adopted a modern, western-based lifestyle no longer grounded in the traditional belief systems. More detailed inquiry is needed, therefore, to assess the influence of culture and religious beliefs on the reporting of PSQ symptoms in Timor Leste and other transcultural settings.

The limitations of the study need to be acknowledged. The study was cross-sectional, cautioning against drawing causal inferences, for example, between PTE exposure and psychotic-like symptoms [12,39]. The traumas measured were limited to the experiences of mass conflict and natural disasters. As noted, we omitted the item for rape, and this may have attenuated the link between trauma and PSQ screen-positive cases. Nor did we assess early trauma such as childhood sexual abuse, or personality vulnerabilities that may be relevant to the development of psychotic-like symptoms. We do not know what the PSQ screen-positive rate was prior to the conflict in Timor Leste. It also is possible that psychotic-like symptoms preceded trauma exposure in some instances. Nevertheless, the chronology recorded for the list of trauma experiences suggests that the vast majority of these events occurred during the Indonesian occupation (1975-1999) and the humanitarian emergency (1999), that is, at least four years prior to participants completing the PSQ.

Consistent with past research, the HTQ community threshold for PTSD would have identified a wider range of persons as symptomatic than a structured interview assessment conducted by clinicians applying DSM-IV criteria [18,27]. Even so, the prevalence of PTSD assessed by the HTQ was relatively low in comparison to other post-conflict settings, although within the range of past epidemiological studies undertaken around the world [24]. In that regard, it is noteworthy that conditions in Timor Leste at the time of the study were consistent with those generally associated with lower rates of PTSD in post-conflict settings, particularly the removal of the source of the terror with the expulsion of the Indonesian military, the restoration of peace by the UN, and the passage of some four years since the conflict [24]. An additional consideration is that some studies have suggested that the presence of PTSD may result in increased reporting of past trauma, although that assertion has been challenged by a recent re-analysis of the trauma histories of combat veterans [40].

## Conclusions

In summary, our findings support the view that post-conflict mental health research needs to extend its focus to psychiatric outcomes beyond PTSD and depression [24,41]. Our results suggest the value of pursuing the assessment of psychotic-like symptoms and their association with trauma in post-conflict settings. Further research also is needed to examine the implications of psychotic-like symptoms in post-conflict settings such as Timor Leste, the extent to which cultural factors and ongoing tensions in the community influence endorsement of these phenomena, and the relationship of symptoms to disabling forms of clinical psychosis.

## Abbreviations

CI: Confidence interval; DSM-IV: Diagnostic and Statistical Manual of Mental Disorders, version four; HTQ: Harvard Trauma Questionnaire; ICD-10: International Classification of Diseases; OR: Odds ratio; PSQ: Psychosis Screening Questionnaire; PTSD: Post-traumatic stress disorder; SCID: Structured Clinical Interview for the Diagnostic and Statistical Manual of Mental Disorders; UN: United Nations; UK: United Kingdom; WWII: World War Two.

## Competing interests

The authors declare that they have no competing interests.

## Authors’ contributions

IS initiated the focus on psychotic-like symptoms in the study and contributed to training of field personnel, data collection and analysis, and the writing of the manuscript. DS took the lead in designing the overall study, obtaining funding and overseeing all aspects of the data collection, analysis and writing of the manuscript. CBS was the in-country team leader who oversaw the training and supervision of the field team and the process of data collection, management and analysis. ZS provided strategic input into the design of the study, the obtaining of funding and the analysis of the data. PB provided guidance, supervision and support, including expert advice on data analysis and writing of the manuscript. PBJ provided expert advice in conceptualizing the study and provided input into the approach to analysis and to the development of the manuscript. TC advised on the design of the study, undertook most of the analyses and advised on interpretation and writing up of the results. LI played a key role in conceptualizing and supporting the statistical analyses. CM provided assistance in all aspects of the development of the manuscript, overseeing its organization and production. All authors read and approved the final manuscript.

## Pre-publication history

The pre-publication history for this paper can be accessed here:

http://www.biomedcentral.com/1471-244X/12/229/prepub
